# Redefining skin toxicity in the age of precision immunotherapy

**DOI:** 10.1111/jdv.70403

**Published:** 2026-04-25

**Authors:** Florentia Dimitriou, Christian Posch

**Affiliations:** ^1^ Department of Dermatology Inselspital, Bern University Hospital Bern Switzerland; ^2^ Faculty of Medicine University of Bern Bern Switzerland; ^3^ Department for Dermatology, Vienna Healthcare Group Clinic Hietzing Vienna Austria; ^4^ Department of Dermatology and Allergology Technical University of Munich Munich Germany; ^5^ Faculty of Medicine Sigmund Freud University Vienna Vienna Austria

Cutaneous immune‐related adverse events (cirAEs) are among the most frequent toxicities associated with immune checkpoint inhibitors (ICIs). In this issue, Fattore et al.^1^ review the prevention and management of cirAEs and suggest that these reactions provide important insights into the biology of antitumor immunity, autoimmunity and tissue‐specific immune tolerance.

Clinically, cirAEs exhibit a broad spectrum of phenotypes; the most common manifestations include pruritus and maculopapular rash, followed by eczematous dermatitis, lichenoid eruptions and psoriasis; whereas vitiligo‐like depigmentation is almost exclusively found in melanoma patients treated with anti‐PD1‐based ICIs.^2^ Most cirAEs are grade 1–2 and manageable with topical corticosteroids. Early recognition is crucial, since cirAEs are the most common and often the earliest manifestation of immune toxicity, allowing for prompt intervention and prevention of progression to more severe modalities, exacerbation or chronicity. Severe phenotypes, including Stevens–Johnson syndrome (SJS)/toxic epidermal necrolysis (TEN)‐like reactions, although rare, underscore the potentially life‐threatening spectrum of these toxicities, making accurate recognition important for prognostic assessment and treatment initiation. Notably, cirAEs may differ from their idiopathic counterparts. For example, they may preferentially affect sun‐exposed areas, or lack classical clinical hallmarks.

Current mechanistic concepts that contextualize the broad spectrum of cirAEs include disruption of peripheral immune tolerance, activation of self‐reactive T‐cells against skin antigens, cytokine storm and production of autoantibodies that lead to complement activation.^3^ Lichenoid eruptions are characterized by cytotoxic CD8^+^ T‐cell accumulation at the dermoepidermal junction, keratinocyte apoptosis and upregulation of Th1‐associated cytokines and chemokines; Goldinger et al.^4^ described that this interface dermatitis can resemble SJS/TEN, yet with gene expression profiles distinct from acute graft‐versus‐host disease, indicating unique immunopathologic endotypes. Reactivation of tissue‐resident memory T‐cells likely increases local inflammation, while polarization toward Th1 and Th17 immune response shapes cirAE phenotypes.^5^ In ICI‐associated bullous pemphigoid, IgG autoantibodies against BP180 trigger complement activation and granulocyte recruitment at the dermal‐epidermal junction.^3^ Vitiligo‐like depigmentation reflects cross‐antigen recognition between melanoma cells and melanocytes, highlighting the narrow margin between desired antitumor immunity and unwanted autoimmunity.^3^ This link highlights an ongoing challenge in immuno‐oncology, namely, uncoupling the therapeutic efficacy from immune‐mediated toxicity.

Fattore et al. also emphasize the need to refine risk stratification. While predictive biomarkers remain elusive, emerging data suggest that tumour entity and patient‐related factors such as age, sex, pre‐existing autoimmune diseases, genetic background, as well as the exposome may modulate susceptibility to the type of toxicity. With regard to the microbiome, skin, as a barrier organ, shares immunological features with the gut, pointing toward a skin–gut axis and a potential role in shaping immunity and, ultimately, cirAE risk. Future research should define how gender‐ and age‐related differences, as well as environmental exposures and medications (e.g. antibiotics) influence cirAE profiles.

A key contribution of the article by Fattore et al. is its emphasis on precise nomenclature and improved grading. The current CTCAE system relies heavily on body surface area (BSA), neglecting symptom burden (e.g. intractable pruritus), lesion morphology, anatomical location, psychological impact and duration or type of immunosuppression required. This lack of granularity may lead to inappropriate treatment interruption or under‐treatment, with significant implications for quality of life (QoL) and tumour control. Persistent or chronic irAEs, especially those requiring prolonged immunosuppression, can profoundly impair QoL, yet these aspects are insufficiently captured in current grading algorithms.

Early dermatologic consultation, biopsy and multidisciplinary collaboration are essential. Although most cirAE cases respond to topical corticosteroids, there is growing momentum toward steroid‐sparing strategies. Biologic agents targeting specific inflammatory pathways, such as IL‐4/13, IL‐17, IL‐23 or TNFα, have shown promise in selected cases. These targeted approaches can mitigate toxicity, but their impact on the antitumor immunity should be explored in prospective studies. Importantly, by observing which pathways drive specific phenotypes and respond to targeted blockade, we can refine our understanding of immune dysregulation and potentially prevent their onset or progression.

The skin is the ideal model organ for translational research in irAEs: It is accessible, highly prevalent as a toxicity site and can serve as a sentinel organ for diverse immunologic phenotypes. Integration of histopathology, molecular profiling, cytokine signatures and microbiome analyses will likely yield new insights for predictive biomarkers and risk‐adapted monitoring. As novel immunotherapies such as T‐cell engagers and CAR‐T cells enter clinical practice, comprehensive dermatologic safety profiling will be essential. The precise understanding and management of cirAEs will be crucial to optimize both patient safety and oncologic outcomes.

## CONFLICT OF INTEREST STATEMENT

FD none; CP declares consulting fees from MERCK, UCB, Incyte, Novartis, Almirall, Astra Zeneca, Boehringer Ingelheim, BMS, MSD, Pierre Fabre, Leo, Lilly, Sanofi, Takeda, Pelpharma, Janssen; honoraria from MERCK, UCB, Incyte, Novartis, Almirall, Astra Zeneca, Boehringer Ingelheim, BMS, MSD, Pierre Fabre, Leo, Lilly, Sanofi, Takeda, Pelpharma, Janssen; travel support from MERCK, UCB, Incyte, Novartis, Almirall, Astra Zeneca, Boehringer Ingelheim, BMS, MSD, Pierre Fabre, Leo, Lilly, Sanofi, Takeda, Pelpharma, Janssen; all unrelated to the current work.

## Data Availability

Data sharing not applicable to this article as no datasets were generated or analysed during the current study.
